# Scientific Fallacies

**Published:** 1884-11

**Authors:** 


### Scientific Fallacies.

We believe it was old Dr. Johnson who said: "Let any fool, preaching no matter how absurd a doctrine, address a crowd at the street corner, and he will be sure to find some disciples."

So it seems to be to-day in the world of science. Any one who claims to have made a discovery, and on the strength of it brings out a novel theory, immediately finds a train of disciples drawn from the crowd of those who, like the Athenians of yore, are ever anxious to see and hear some new thing.

An instance in point is Dr. Koch's famous bacillus theory. With it he captured the scientific world as one captures a gudgeon with a fly. What is the result? Where is the theory after two or three short years of testing? Does any one now advocate the existence of a tubercle microbe as a factor of any consequence, either in the treatment or in the prophylaxis of the disease? So it was some years ago, when fevers of all kinds were dosed with enormous quantities of alcoholics, on some general theory of fever.

Most of these ephemeral doctrines arise from the study of physiological therapeutics, from an unhesitating acceptance of the teaching that the only true basis of a rational pharmacy is in experiments on lower animals and on persons in health.

The fallacy of such a doctrine has been so often demonstrated that it is useless again to refer to it. Yet it can not too often be repeated. We can only learn disease from disease. Pathology is not physiology, and is not akin to it. As Virchow has often and ably urged, it is incorrect to make any suppositions about the former from the latter. Their laws and their processes are different.

So it must also be with the effect of various agents. If we wish to learn their effect on disease in man, we must study them in disease in man, and not elsewhere.

If a man empties his purse into his head, no man can take it away from him. An investment in knowledge always pays the best interest.—*Franklin.*

